# Identification of *Toxocara canis* Antigen-Interacting Partners by Yeast Two-Hybrid Assay and a Putative Mechanism of These Host–Parasite Interactions

**DOI:** 10.3390/pathogens10080949

**Published:** 2021-07-28

**Authors:** Ewa Długosz, Małgorzata Milewska, Piotr Bąska

**Affiliations:** 1 Division of Parasitology and Invasive Diseases, Department of Preclinical Sciences, Institute of Veterinary Medicine, Warsaw University of Life Sciences—SGGW, 02-786 Warsaw, Poland; malmilewska9@gmail.com; 2 Division of Pharmacology and Toxicology, Department of Preclinical Sciences, Institute of Veterinary Medicine, Warsaw University of Life Sciences—SGGW, 02-786 Warsaw, Poland; piotr_baska@sggw.edu.pl

**Keywords:** toxocariasis, host–parasite interactions, yeast two-hybrid assay

## Abstract

*Toxocara canis* is a zoonotic roundworm that infects humans and dogs all over the world. Upon infection, larvae migrate to various tissues leading to different clinical syndromes. The host–parasite interactions underlying the process of infection remain poorly understood. Here, we describe the application of a yeast two-hybrid assay to screen a human cDNA library and analyse the interactome of *T. canis* larval molecules. Our data identifies 16 human proteins that putatively interact with the parasite. These molecules were associated with major biological processes, such as protein processing, transport, cellular component organisation, immune response and cell signalling. Some of these identified interactions are associated with the development of a Th2 response, neutrophil activity and signalling in immune cells. Other interactions may be linked to neurodegenerative processes observed during neurotoxocariasis, and some are associated with lung pathology found in infected hosts. Our results should open new areas of research and provide further data to enable a better understanding of this complex and underestimated disease.

## 1. Introduction

Human toxocariasis is a neglected parasitic disease caused by the dog and cat roundworms—*Toxocara canis* and *Toxocara cati,* respectively. A systematic review and meta-analysis of the literature performed recently led to the estimation that almost one fifth (19%) of the world’s human population is seropositive for *Toxocara* larvae; the highest seroprevalence rates were found in Africa (37.7%) and the lowest in the Eastern Mediterranean region (8.2%) [1]. In Central Europe, the seroprevalence varies from 1.5% to 30%, with the Czech Republic representing the average (3.6%) [2] and a rate of 14.5% in teenagers in Poland [3].

Upon infection, larvae migrate in the human host leading to location-dependent clinical syndromes. Visceral larva migrans (VLM) is associated with hepatic and pulmonary larval migration and results in abdominal pain, fever, coughing, wheezing, asthma and hepatomegaly. Ocular larva migrans (OLM), caused by larval migration into the eye, results in uveitis or endophthalmitis that can lead to a loss of sight. Neurotoxocariasis is caused by the presence of larvae in the brain and in rare cases may lead to neurological disorders but can also cause subtle effects on cognition in children [4]. In comparison, the syndrome that is most common, but also most difficult to diagnose, is covert toxocariasis, featuring nonspecific symptoms such as arthralgia, lymphadenopathy, fever or headaches [5]. *Toxocara* spp. also contribute to the development of allergic diseases, including asthma, chronic urticaria or angioedema [6,7,8].

A limitation to our understanding of the aetiology and progression of disease, or lack thereof, is an insufficient understanding of the host–parasite interactions. Recently, a draft genome of *T. canis* identified 870 excretory-secretory (ES) proteins putatively involved in host invasion and in host–parasite interactions, such as immune evasion and/ or immune modulation [9]. These proteins include proteases, cell adhesion molecules, lectins, SCP/TAPS proteins and mucins. However, very little is known about host molecules that interact with the abovementioned antigens. To address this knowledge gap, we used the yeast two-hybrid (Y2H) assay to screen a human cDNA library and analyse the interactome of *T. canis* larval molecules.

Y2H has been used to predict interacting partners of many parasitic molecules, including those of *Schistosoma mansoni* histone deacetylase 8 [10], *Ehrlichia chaffeensis* TRP32 [11] and *Toxoplasma gondii* MIC2 [12]. In our study, three molecules highly expressed by *T. canis* larvae were chosen as bait: Tc-MUC-3, Tc-CTL-1 and Tc-TES-26. Tc-MUC-3 is a component of TES-120 O-methylated glycoproteins that contain mucin and ShK/SXC domains. These molecules build the surface coat of the larvae but are also secreted by the parasite [13]. Tc-CTL-1 is a 32 kDa C-type lectin [14] that is thought to interfere with infiltration of host leukocytes by competitive inhibition of selectin-mediated inflammation [15]. Tc-TES-26 is a 26 kDa phosphatidylethanolamine binding protein [16]. This protein retains a hydrophobic motif thought to mediate lipid binding and, like mucins, contains two ShK/SXC domains. These proteins are constituents of *Toxocara* excretory-secretory products (TES) [17] and, coupled with their high expression profile, represent strong candidates to interact with host molecules. Using these proteins as bait we performed the Y2H assay, which allowed us to predict numerous host–parasite interactions.

## 2. Results

### 2.1. Construction of Bait Plasmids

Tc-MUC-3, Tc-CTL-1 and Tc-TES-26 coding sequences lacking signal peptides were amplified on the template of *T. canis* larvae cDNA. All three sequences were cloned into pGBKT7 DNA-BD plasmids using *Eco*RI and *Bam*HI restriction sites. Successful cloning was confirmed by Sanger sequencing.

### 2.2. Autoactivation and Toxicity Test

None of the three bait Y2HGold transformants showed autoactivation activity as indicated by the growth of white colonies on SD/-Trp and SD/-Trp/X plates and absence of colonies on SD/-Trp/X/A plates. Moreover, the colonies encoding bait were the same size as those containing empty pGBKT7 DNA-BD vector on SD/-Trp plates, indicating that the bait were not toxic to yeast.

### 2.3. Yeast Two-Hybrid Screen

The mating efficiency was at least 2% in all three screens, indicating that over 1 million diploids were screened in each case. False positive interactions were eliminated by co-transformation of yeast with each prey plasmid together with the bait-containing plasmid and empty pGBKT7 plasmid and selection of co-transformants on QDO/X/A agar plates. Prey which activated reporter genes without the presence of bait were eliminated. Finally, prey from true positive interactions were sequenced and analysed using BLASTn and BLASTx search.

### 2.4. Identification of Interacting Proteins

In total, we identified 16 positive interactions, 9 with Tc-MUC-3, 5 with Tc-CTL-1 and 2 with the Tc-TES-26 bait. All identified prey are listed in Table 1.

**Figure 1:**
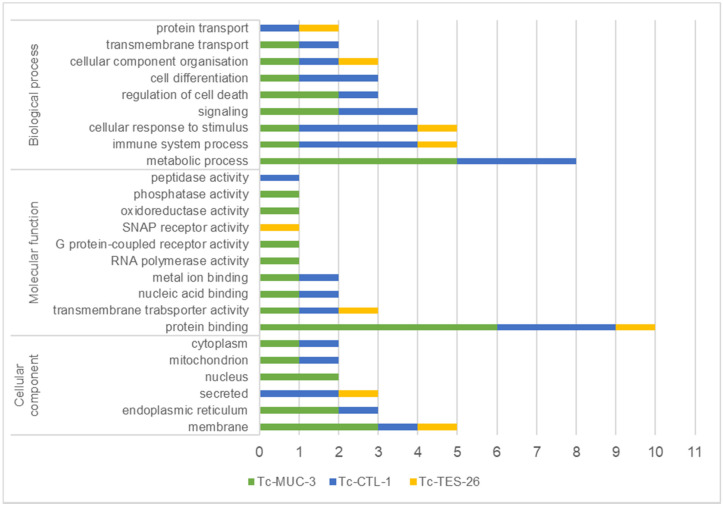
*T. canis* interacting proteins identified by Y2H, classified by predicted biological/molecular function and cellular component according to gene ontology (GO). Classification was obtained from UniProtKB. The x-axis number represents the number of identified proteins classified to each category.

Based on gene ontology (GO) annotations, proteins were classified by predicted biological process, molecular function and cellular component (Figure 1).

Target proteins were associated with a broad range of biological processes such as metabolism, immune system function, response to stimuli, signalling, regulation of cell death, cell differentiation, cellular component organisation and transport. The molecular functions of the identified prey were associated with protein-, ion- and nucleic acid-binding, transmembrane transporter activity, oxidoreductase, phosphatase and peptidase activity as well as receptor activity. Target proteins interacting with *T. canis* molecules were predominantly localized in the plasma membrane (29%) and endoplasmic reticulum (18%) or were secreted proteins (18%). The remaining target proteins were localized in the nucleus, mitochondria and cytoplasm. The full list of GO annotations is presented in the Supplementary Materials (Supplementary File S1).

**Figure 2:**
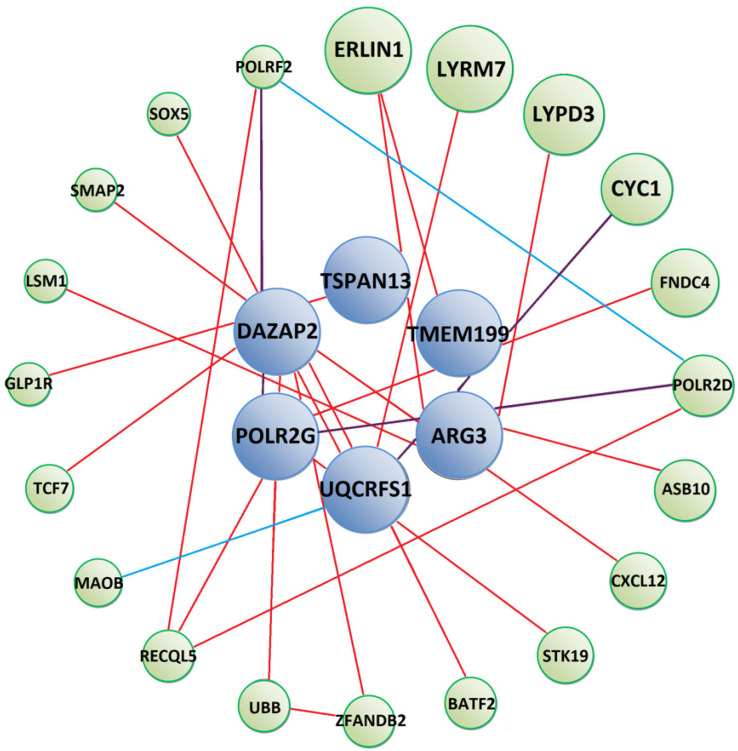
Predicted protein–protein interaction network between Tc-MUC-3 interacting prey (blue circles) and other human proteins (green circles). Red lines show physical interactions, blue lines show proteins which share a common pathway and purple lines represent both types of interactions. Detailed information on interacting proteins is included in Supplementary Materials (Supplementary File S2).

**Figure 3:**
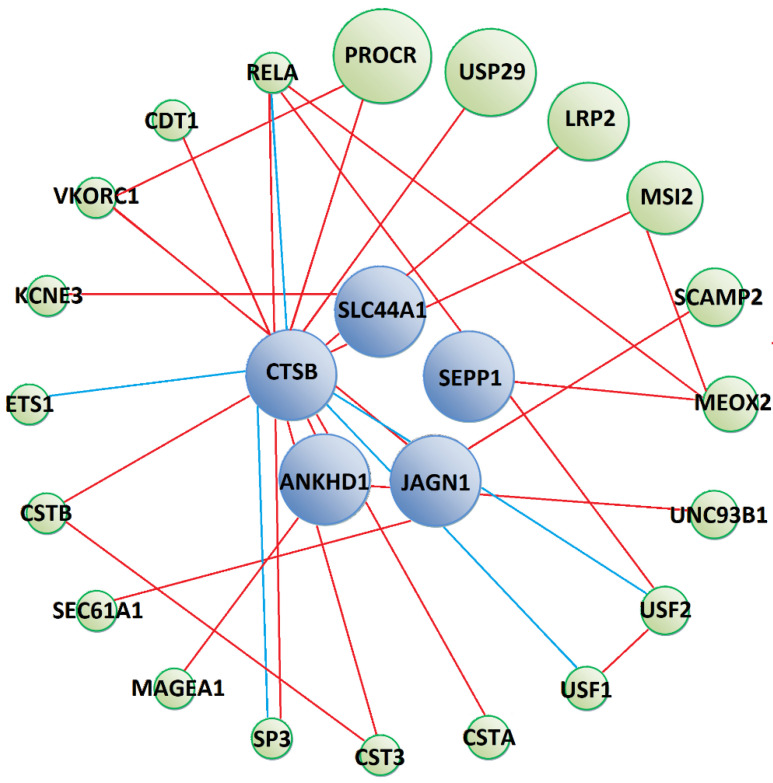
Predicted protein–protein interaction network between Tc-CTL-1 interacting prey (blue circles) and other human proteins (green circles). Red lines show physical interactions, blue lines show proteins which share a common pathway and purple lines represent both types of interactions. Detailed information on interacting proteins is included in Supplementary Materials (Supplementary File S3).

**Figure 4:**
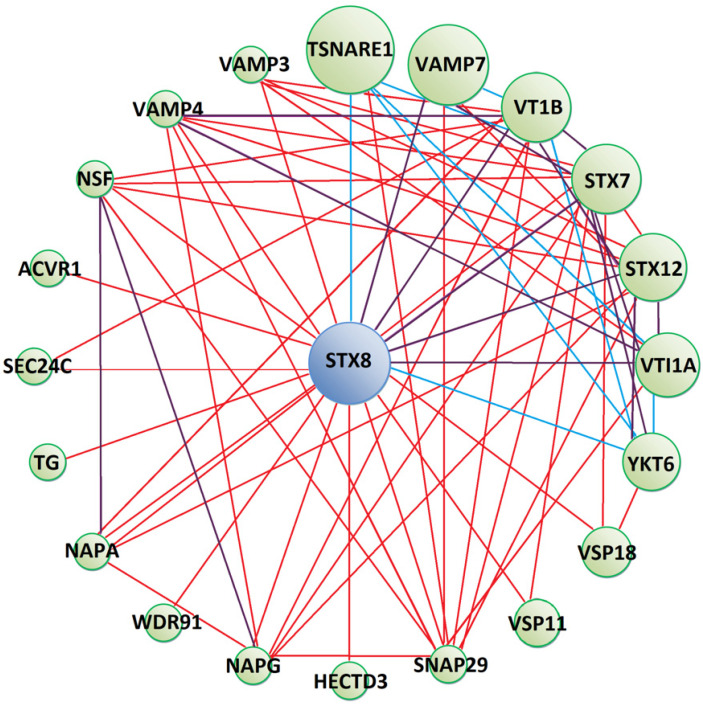
Predicted protein–protein interaction network between Tc-TES-26 interacting prey (blue circle) and other human proteins (green circles). Red lines show physical interactions, blue lines show proteins which share a common pathway and purple lines represent both types of interactions. Detailed information on interacting proteins is included in Supplementary Materials (Supplementary File S4).

To further analyse possible mechanisms of *T. canis* interactions with human targets, we constructed protein–protein interaction networks between our identified prey and other human proteins (Figure 2, Figure 3 and Figure 4). Tc-MUC-3 prey interact with proteins involved in immune response (FNDC4, CXCL12, BATF2, TCF7), mitochondrial respiratory chain (LYRM7, CYC1), ubiquitin-mediated protein degradation (ASB10, ZFANDB2, UBB), signalling pathways (STK19, GLP1R, ZFANDB2, UBB), mRNA synthesis (POLR2D, RECQL5, LSM1, POLRF2) and cholesterol homeostasis (ERLIN1) (Figure 2). Detailed information on interacting proteins is shown in the Supplementary Data (Supplementary File S2). Moreover, GeneMania was used to predict that POLR2G participates in a common pathway with other RNA polymerase II subunits, and UQCRFS1 was found to share a pathway with monoamine oxidase B (MAOB). The participation of the two proteins in the Huntington’s disease pathway was found using bioinformatics.

Prey that interacted with Tc-CTL-1 were predicted to form physical interactions with human proteins involved in signalling processes (PROCR, LRP2, MAGEA1), protein transport (SCAMP2, SEC61A1), immune response (UNC93B1, CTS3, CSTB, SP3, RELA) (Figure 3) and other biological processes, which are detailed in the Supplementary Materials (Supplementary File S3). Moreover, GeneMania predicted CTSB prey to participate in common pathways with several transcription factors (USF1, USF2, SP3, ETS1 and RELA), some of which are important regulators of the immune response (SP3, ETS1 and RELA). However, these interactions were not substantiated by KEGG Pathway database analysis.

Syntaxin 8 (STX8), a protein interacting with Tc-TES-26, is anticipated to form protein networks with molecules mostly involved in vesicle-mediated transport (Figure 4). These proteins possess Soluble NSF Attachment Proteins (SNAP) receptor activity. Apart from physical interactions, YKT6, SNAP29, STX7, STX8, VAMP3, CAMP4, VAMP7, VTI1A and VTI1B proteins were predicted to participate in SNARE (SNAP Receptor) interactions in vesicular transport pathways, as indicated by KEGG Pathway database analysis. Moreover, SNAP29, VTI1A and VTI1B take part in exocytosis and regulate the degranulation of immune cells. SEC24C and VAMP3 take part in antigen processing and presentation by MHC I and II and VTI1A, VSP18 and VSP11 in the process of autophagy. Detailed information on proteins forming the interaction network is shown in Supplementary Materials (Supplementary File S4).

## 3. Discussion

**Figure 5:**
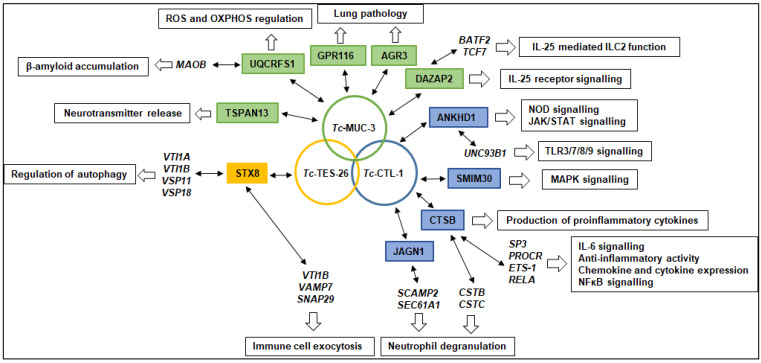
Hypothetical mechanisms of host–parasite interactions and their contribution to the pathology of toxocariasis. *T. canis* larval molecules are shown in circles; predicted interacting prey proteins identified using the Y2H assay are shown in colour boxes; proteins interacting with prey proteins identified using bioinformatics are shown in italics; processes that may be affected by selected molecules are shown in open boxes.

Screening of a human cDNA library led to the identification of 16 putative interacting partners for the three *T. canis* molecules in this study. GO analyses revealed many different molecular functions and biological processes in which these proteins may be involved. We also searched for human molecules predicted to form secondary interactions with prey proteins. It is believed that the primary role of TES products is manipulating, blocking and/or evading immune responses of the host. Therefore, we focused on the most important interactions that may provide new insights into the immunopathology of human toxocariasis, as summarized in Figure 5.

Some of the identified prey proteins are involved in signalling processes. The Tc-MUC-3 interacting molecule, DAZ associated protein 2 (DAZAP2), is a negative regulator of interleukin 25 (IL-25) signal transduction. This protein binds to the IL-25 receptor, IL17RB, and its degradation by ubiquitination is an essential step for commencement of IL-25 signalling [18,19]. IL-25 is a cytokine which initiates a proallergic Th2 response in the airways [20]. The development of a Th2 response during toxocariasis was observed in several studies [21,22,23]. Our previous study showed that recombinant *T. canis* mucins stimulate the production of Th2 cytokines IL-4 and IL-5 by splenocytes from infected mice [24]. Mucin could interact with DAZAP2 to abolish its negative regulatory activity, leading to excessive activation of IL-25 receptor and promotion of a Th2 response.

Our protein interaction network analysis suggested that DAZAP2 forms physical interactions with other molecules involved in immune response regulation, CXCL12 chemokine, which is strongly chemotactic for lymphocytes [25], and with BATF2 and TCF-7, transcription factors which stimulate Th2 differentiation [26,27,28].

Ankyrin repeat and KH domain containing 1 protein (ANKHD1), which was identified as a putative Tc-CTL-1 interacting partner, is also involved in signalling and has been shown by Y2H, to interact with nucleotide-binding oligomerization domain containing 2 (NOD2), which is one of the major receptors in the innate immune response activating NF-κB and MAPK signalling pathways [29]. Moreover, ANKHD1 is a positive regulator of Janus Kinase and Signal Transducer and Activator of Transcription (JAK/STAT) signalling pathway and controls the levels of a subset of pathway receptors in human cells [30]. ANKHD1 also forms interactions with UNC93B1, which is involved in Toll-like receptor (TLR) 3/7/8/9 signalling [31]. Binding of Tc-CTL-1 to ANKHD1 may affect the signal transduction in NOD2, JAK/STAT and TLR pathways.

The third protein involved in signalling is the small integral membrane protein 30 (SMIM30), which is a small endogenous peptide encoded by a small open reading frame (smORF) present in LINC00998 long non-coding RNA that participates in activation of the MAPK signalling pathway in hepatocellular carcinoma cells [32].

Some of the proteins identified in this study were shown to affect the production of cytokines. Of note is the solute carrier 44A1 (SLC44A1; CD92), a Na^+^-independent choline transporter present in the cellular as well as mitochondrial membrane [33]. SLC44A1 has a role in immune cell function as part of an auto-regulatory signalling loop that controls the expression and maintenance of IL-10 production in dendritic cells [34] and THP-1 cells [35]. Inhibition of this transporter in lipopolysaccharide (LPS)-treated dendritic cells, with use of a monoclonal antibody, augmented the LPS-induced IL-10 production [34]. In our previous study, we made a similar observation; the production of IL-10 from LPS stimulated THP-1 cells increased after additional treatment with *T. canis* molecules [36]. Tc-CTL-1 interaction with SLA44A1 is, thus, an important topic for future experiments.

Cathepsin B (CTSB) belongs to a family of lysosomal cysteine proteases and plays an important role in intracellular proteolysis. It is also necessary for production of inflammatory cytokines, such as TNF-α [37]. CTSB is also involved in the activation of the NLRP3 inflammasome and IL-1β production [38]. As shown in our protein–protein interaction networks, CTSB interacts with transcription factors regulating the production of cytokines, Specificity protein 3 (SP3) [39], Protein C-ets-1 (ETS-1) [40] and p65 (RELA) [41]. Our Y2H assay identified an interaction between Tc-CTL-1 and CTSB. This binding might lead to loss of CTSB function and inhibition of proinflammatory cytokine secretion. Such inhibition of TNF-α and IL-1β expression in mice brains infected with *T. canis* was recently shown [42]. These authors conclude that such downmodulation of proinflammatory cytokines enables the survival of the parasite as well as the neuro-infected paratenic host. Other studies agree with this observation and have shown that macrophages and splenocytes from mice infected with *T. canis* produce lower amounts of TNF-α compared to uninfected controls [24,43].

CTSB also forms physical interactions with cysteine proteinase inhibitors—cystatins (CST-A, -B and -C). Of these three, CST3 (cystatin C) is especially involved in the regulation of the immune response [44], but, as shown by gene ontology analysis, both CSTB and CSTC may be involved in neutrophil degranulation. Another Tc-MUC-3 interacting protein, jagunal homolog 1 (JAGN1), is critical for the differentiation and maintenance of human neutrophils [45]. In mice, deletion of *Jagn1* leads to an impaired formation of cytotoxic granules, as well as defective myeloperoxidase (MPO) release [46]. JAGN1-silenced neutrophils remain capable of producing NETs but are ineffective in killing pathogens due to the altered MPO expression [47]. Our protein network analysis indicates that JAGN-1 interacts with two proteins involved in protein transport, both of which may participate in the process of neutrophil degranulation, secretory carrier-associated membrane protein 2 (SCAMP2) [48] and transport protein Sec61 subunit alpha isoform 1 (SEC61A1) [49].

As mentioned above, *T. canis* molecules may affect neutrophil function. During infection, inflammatory infiltrate around larvae consists mainly of neutrophils and eosinophils [50]. As high neutrophil counts follow the migration of larvae through the organs and tissues, the authors speculate that these cells may act as the first line of defence against *T. canis* larvae. Another study, however, showed that despite neutrophil and macrophage adherence to the larval surface, this attachment does not result in the degranulation and causes no damage to the worms [51]. The mechanisms underlying neutrophil hyporesponsiveness remain unknown, but the results of our study suggest that Tc-CTL-1 interaction with the abovementioned human molecules may play a role.

Moreover, the interaction of Tc-TES-26 with Syntaxin 8 (STX8), belonging to the t-SNARE (SNAP receptor on target membrane) superfamily of proteins, which are involved in vesicle trafficking and docking [52], may also interfere with the process of exocytosis in immune cells. Among other molecules, STX8 is required for efficient lytic granule trafficking in cytotoxic T lymphocytes [53] and for the secretion of dense granules by platelets [54]. As shown by our protein interaction networks and GO analysis, proteins interacting with STX8 (VAMP7, VTI1B, SNAP29) are involved in exocytosis and regulation of degranulation of immune cells [55].

Two proteins identified in the study are responsible for ion binding, UQCRFS1 and Selenoprotein P (SEPP1). SEPP1 was identified as a Tc-CTL-1 interacting molecule. This protein participates in selenium transport but also shows anti-oxidant properties [56]. Deletion of *Sepp1* leads to decreased whole-body selenium levels [57]. This may be beneficial for some parasites, as a selenium deficient diet reduces resistance to *Heligmosomoides polygyrus* infection [58], and selenium supplementation enhances the protective response against *T. canis* larvae in mice [59]. Therefore, Tc-CTL-1 interaction with SEPP1 may interfere with selenium distribution, decreasing selenium levels in the host, possibly promoting parasite survival.

*T. canis* infection leads to disturbances in lung function and is considered as a possible etiological agent of asthma [8,60]. Respiratory distress, such as wheezing and coughing, can be caused by the migration of larvae through the lungs [61]. Experimental studies have shown that *T. canis* infection results in chronic pulmonary inflammation with perivascular infiltration, mainly consisting of eosinophils and lymphocytes and induction of a dominant Th2-type immune response [62]. Additionally, *T. canis* infection leads to tracheal inflammation, thickening of the tracheal smooth muscle layers, narrowing of trachea, airway hyperresponsiveness and lung mucus hypersecretion, eventually leading to the development of asthma-like symptoms [23,60]. We, and others, have shown that *T. canis* induces high levels of IgE that are specific to the TES antigens but also to particular mucins [62,63].

Some of the identified *Toxocara* target proteins may be involved in the pathological processes observed in the lungs of infected hosts. For instance, DAZAP2 may contribute to development of the Th2-type immune response. A putative Tc-MUC-3 interacting partner, adhesion G protein-coupled receptor F5 (ADGRF5 or GPR116), regulates pulmonary alveolar homeostasis and its knockout leads to an early accumulation of surfactant in the lungs of mice, followed by a massive infiltration of foamy alveolar macrophages, eventually progressing into an emphysema-like pathology [64,65]. Dysfunctions in pulmonary surfactant systems are associated with severe respiratory pathologies [66]. It is possible that larval molecules block the receptor activity leading to pathological reactions in the lung.

The third protein involved in airway function is anterior gradient 3 (AGR3), a disulphide isomerase family member, which is expressed in the ciliated cells in the airway epithelium. Mice lacking AGR3 display a lower beat frequency compared with control mice [67]. Compromised mucociliary clearance contributes to changes in relative proportions and viscosity of host mucins, which, in turn, contribute to airway obstruction [68]. AGR3 was shown to interact with mucins [69] and this may explain its binding to Tc-MUC-3, which is a mucin-like glycoprotein [13]. It remains to be confirmed if, and to what extent, this interaction may contribute to the pathology of toxocariasis.

Neurotoxocariasis is another important issue concerning *T. canis* infection. The location of larvae in the central nervous system manifests as encephalopathy with cognitive decline, meningoencephalitis, cerebral vasculitis, epilepsy, visual impairment, myelitis, radiculitis, cranial nerve involvement or skeletal muscle affection [70]. One Tc-MUC-3 binding partner, tetraspanin 13 (TSPAN-13), may contribute to the pathology in neurotoxocariasis through regulation of Ca^2+^ channel activity in defined synaptic membrane compartments, influencing the release of neurotransmitters [71]. Tc-MUC-3 also interacts with Rieske iron-sulphur protein (UQCRFS1). This molecule together with cytochrome b and cytochrome c1 are three highly conserved subunits which contain redox active centres and are responsible for the catalytic activity of ubiquinol-cytochrome c oxidoreductase (E.C. 1.10.2.2, also known as Complex III), a central component of the mitochondrial respiratory chain [72]. UQCRFS1 knockout results in increased intracellular level of free oxygen radicals (ROS), and assembly of oxidative phosphorylation (OXPHOS) complexes is impaired in the absence of this molecule [73]. Mitochondrial dysfunctions, OXPHOS and ROS production disturbances contribute to neurodegenerative processes [74].

Furthermore, KEGG Pathway analysis suggests that UQCRFS1 is involved in a common pathway with amine oxidase B (MAOB). MAOB regulates the expression of β-amyloid, which is a key molecule in the pathogenesis of Alzheimer’s disease [75]. MAOB may also be involved in the pathology of neurotoxocariasis, as increased levels of β-amyloid precursor protein [76] and insoluble β-amyloid accumulations were observed in the brains of mice infected with *T. canis* [77].

Autophagy is another process affecting the homeostasis in the nervous system. Autophagy dysfunctions may contribute to cognitive defects and stress-induced mood disorders by compromising adult neurogenesis [74]. Astrocytes treated with TES have been shown to lead to increased cellular mortality; cells undergo apoptosis while the process of autophagy is limited [78]. As we have shown, STX8 is predicted to interact with numerous molecules responsible for the regulation of autophagy: VAMP3, VAMP7, STX7 and VTI1B [79]. Interactions between TES molecules and STX8 may therefore affect the formation of protein complexes important for the process of autophagy, and this could contribute to the pathology of neurotoxocariasis.

To conclude, we have shown putative interactions between human proteins and three molecules secreted by *T. canis* larvae. We have also discussed how these predicted host–parasite interactions may contribute to the pathology of infection in the human host. This should open new areas and provide further ideas in the research field of toxocariasis and enable a better understanding of this complex and underestimated disease.

## 4. Materials and Methods

### 4.1. Bait Plasmid Construction

RNA isolation from *T. canis* larvae was performed as previously described [24]. One microgram of total RNA was reverse-transcribed into cDNA using Thermo Scientific RevertAid H Minus Reverse Transcriptase (Thermo Fisher Scientific, Waltham, MA, USA) according to manufacturer’s instructions. Bait plasmids were constructed by cloning cDNA fragments encoding Tc-MUC-3 (AF167708), Tc-CTL-1 (AF041023) and Tc-TES-26 (U29761) mature proteins in frame into pGBKT7 DNA-BD vectors (Clontech Laboratories, Inc. A Takara Bio Company, Mountain View, CA, USA). The coding fragments were PCR amplified using primers shown in Table 2 and cloned using *Eco*RI and *Bam*HI restriction sites.

### 4.2. Yeast Transformation, Autoactivation and Toxicity Test

Each bait construct was transformed into the *Saccharomyces cerevisiae* Y2HGold strain (Clontech Laboratories, Inc.) according to the instructions of Yeastmaker Yeast Transformation System 2 (Clontech Laboratories, Inc.). In parallel, control vectors supplied with the Matchmaker Gold yeast two-hybrid system were transformed into yeast; pGBKT7-53 and pGBKT7-Lam into Y2HGold and pGADT7-T into Y187. Transformants were selected on tryptophan-free SD medium (SD/-Trp) for 3–5 days.

To test the bait for autoactivation colonies containing bait as well as positive and negative controls supplied with the MatchmakerGold, yeast two-hybrid systems were plated on SD/-Trp supplemented with 40 µg/mL X-α-Gal (SD/-Trp/X) and SD/-Trp supplemented with 40 µg/mL X-α-Gal and 125 ng/mL Aureobasidin A (SD/-Trp/X/A) agar plates and grown for 3–5 days. Lack of autoactivation was indicated by the growth of white colonies on SD/-Trp and SD/-Trp/X plates and the absence of colonies on SD/-Trp/X/A plates.

To test the bait for toxicity, the colonies containing bait along with the colonies containing empty pGBKT7 DNA-BD vector were spread on SD/-Trp plates and grown for 3–5 days. Lack of toxicity was indicated by comparing the sizes of the bait colonies with the colonies containing an empty vector.

### 4.3. Yeast Two-Hybrid Screen

The yeast two-hybrid screen was performed using Matchmaker Gold Yeast Two-Hybrid System (Clontech Laboratories, Inc. Co 630489). This system included yeast strains, media and reagents used for the screen and the procedure of the yeast two-hybrid screen was preformed according to the manufacturer’s instructions. Each Y2HGold strain containing the particular bait was mated with Y187 *S. cerevisiae* transformed with universal human cDNA library (Clontech Laboratories, Inc. Co 630480). Control reactions were performed: Y187[pGADT7-T] was mated with either Y2HGold[pGBKT7-53] or Y2HGold[pGBKT7-Lam] to obtain positive and negative interaction controls, respectively. The transformed cultures were plated on double dropout SD medium lacking leucine and tryptophan supplemented with 40 µg/mL X-α-Gal and 125 ng/mL Aureobasidin A (DDO/X/A). The plates were incubated at 30 °C for 3–5 days. All blue colonies were then patched out onto higher stringency agar plates containing quadruple dropout medium lacking adenine, histidine, leucine and tryptophan, supplemented with 40 µg/mL X-α-Gal and 125 ng/mL Aureobasidin A (QDO/X/A) and incubated at 30 °C for 3–5 days. Blue colonies of normal size were segregated three times on double dropout SD medium containing X-α-Gal (DDO/X).

In order to identify prey, yeast colony PCR was performed on all blue colonies obtained after segregation using Matchmaker Insert Check PCR Mix 2 (Clontech Laboratories, Inc., no. 630497). Similarly sized PCR products were then analysed by restriction digestion using *Bsu*RI to eliminate duplicate clones.

In the next step, prey plasmids were rescued from yeast colonies using Easy Yeast Plasmid Isolation Kit (Clontech Laboratories, Inc. no. 630467), transformed into *Escherichia coli* competent cells and selected on LB agar plates containing 100 µg/mL ampicillin.

Positive interactions were then confirmed by co-transformation of each rescued prey plasmid with the bait plasmid and empty pGBKT7 DNA-BD as control into Y2HGold. Again, pGBKT7-53 or pGBKT7-Lam vectors were co-transformed with pGADT7-T to obtain positive and negative interaction controls. Transformants were grown on DDO/X and QDO/X/A plates for 3–5 days. True positive hits were identified by observation of blue colonies containing the bait and the candidate prey on DDO/X and QDO/X/A plates on the condition that transformants containing the same candidate prey and empty pGBKT7 DNA-BD plasmid formed white colonies on DDO/X plates and no colonies on QDO/X/A plates.

### 4.4. Positive Prey Analysis by Bioinformatics

Prey plasmids were analysed by Sanger sequencing. Obtained sequences were analysed by BLASTn and BLASTx search to identify the corresponding genes. Identified proteins were classified according to their predicted molecular function, biological process and cellular component using the UniProtKB database [80]. Proteins physically interacting, or sharing biological pathways with prey, were identified using GeneMania [81]. Pools of genes (prey and interacting proteins) were analysed for enrichment in biological pathways from the KEGG database using the DAVID functional annotation tool [82]. The pathways identified with *p* value < 0.05 were considered as positive.

## Figures and Tables

**Table 1:** List of bait-interacting proteins identified in the study. Protein acronyms are shown in brackets.

Bait	Interacting Protein	NCBI ID	UniProt ID
Tc-MUC-3	Tetraspanin 13 (TSPAN13)	NM_014399.3	O95857
	Small integral membrane protein 30 (SMIM30)	NM_001352688.1	A4D0T7
	RNA polymerase II subunit G (POLR2G)	NM_002696.2	P62487
	Adhesion G protein-coupled receptor F5 (ADGRF5)	NM_015234.4	Q8IZF2
	Ubiquinol-cytochrome c reductase, Rieske iron-sulphur polypeptide 1 (UQCRFS1)	NM_006003.2	P47985
	Anterior gradient 3, protein disulphide isomerase family member (AGR3)	NM_176813.4	Q8TD06
	DAZ associated protein 2 (DAZAP2)	NM_014764.3	Q15038
	Transmembrane protein 199 (TMEM199)	NM_152464.2	Q8N511
	Protein phosphatase 2C-like domain containing 1data (PP2D1)	NM_001252657.1	A8MPX8
Tc-CTL-1	Solute carrier family 44 member 1 (SLC44A1)	NM_080546.4	Q8WWI5
	Selenoprotein P (SELENOP)	NM_001093726.2	P49908
	Jagunal homolog 1 (JAGN1)	NM_032492.3	Q8N5M9
	Cathepsin B (CTSB)	NM_001317237.1	P07858
	Ankyrin repeat and KH domain containing 1 (ANKHD1)	NM_017747.2	Q8IWZ3
Tc-TES-26	Syntaxin 8 (STX-8)	AF062077.1	Q9UNK0
	Cysteine rich secretory protein 2 (CRISP)	NM_001142435.3	P16562

**Table 2:** Sequences of linker primers used in the study. Restriction sites are underlined.

Construct	Primer Sequence	Restriction Site
pGBKT7-muc-3	F: CGGAATTCCAATCGATATTCGCAGCA	*Eco*RI
	R: CGGGATCCCGAACAAAAACCGCACGA	*Bam*HI
pGBKT7-ctl-1	F: CGGAATTCTGCGTCAACAACAATGAC	*Eco*RI
	R: CGGGATCCGAGAGGTCTCTTGCATAC	*Bam*HI
pGBKT7-tes-26	F: CGGAATTCCAACAGTGTATGGACAGC	*Eco*RI
	R: CGGGATCCGGCCTGCGATCGATAGAA	*Bam*HI

## Supplementary Materials

The following are available online at https://www.mdpi.com/article/10.3390/pathogens10080949/s1, Supplementary File S1: GO annotations of identified *T. canis* interacting proteins. Supplementary File S2: Tc-MUC-3 prey interaction proteins. Supplementary File S3: Tc-CTL-1 prey interaction proteins. Supplementary File S4: Tc-TES-26 prey interaction proteins.
